# Identification of phosphoenolpyruvate carboxykinase 1 as a potential therapeutic target for pancreatic cancer

**DOI:** 10.1038/s41419-021-04201-w

**Published:** 2021-10-07

**Authors:** Xiao-ren Zhu, Shi-qing Peng, Le Wang, Xiao-yu Chen, Chun-xia Feng, Yuan-yuan Liu, Min-bin Chen

**Affiliations:** 1 Department of Radiotherapy and Oncology, Affiliated Kunshan Hospital of Jiangsu University, Kunshan, China; 2 Changshu Hospital Affiliated to Nanjing University of Chinese Medicine, Changshu, China; 3 Clinical Research and Lab Center, Affiliated Kunshan Hospital of Jiangsu University, Kunshan, China

**Keywords:** Pancreatic cancer, Oncogenes

## Abstract

Pancreatic cancer is the third leading cause of cancer-related mortalities and is characterized by rapid disease progression. Identification of novel therapeutic targets for this devastating disease is important. Phosphoenolpyruvate carboxykinase 1 (PCK1) is the rate-limiting enzyme of gluconeogenesis. The current study tested the expression and potential functions of PCK1 in pancreatic cancer. We show that *PCK1* mRNA and protein levels are significantly elevated in human pancreatic cancer tissues and cells. In established and primary pancreatic cancer cells, PCK1 silencing (by shRNA) or CRISPR/Cas9-induced PCK1 knockout potently inhibited cell growth, proliferation, migration and invasion, and induced robust apoptosis activation. Conversely, ectopic overexpression of PCK1 in pancreatic cancer cells accelerated cell proliferation and migration. RNA-seq analyzing of differentially expressed genes (DEGs) in PCK1-silenced pancreatic cancer cells implied that DEGs were enriched in the PI3K-Akt-mTOR cascade. In pancreatic cancer cells, Akt-mTOR activation was largely inhibited by PCK1 shRNA, but was augmented after ectopic PCK1 overexpression. In vivo, the growth of PCK1 shRNA-bearing PANC-1 xenografts was largely inhibited in nude mice. Akt-mTOR activation was suppressed in PCK1 shRNA-expressing PANC-1 xenograft tissues. Collectively, PCK1 is a potential therapeutic target for pancreatic cancer.

## Introduction

Abnormal activation of multiple signaling cascades are closely associated with the initiation and progression of pancreatic cancer [1]. Dysregulation and overactivation of tyrosine kinases and serine/threonine kinases pathways are key contributors for pancreatic cancer tumorigenesis and development [2, 3], and are important therapeutic targets for intervention [4–6]. Multiple inhibitors/antibodies targeting EGFR, VEGFR, PDGFR, cyclin-dependent kinases, and Src kinases are in various phases of clinical trials testing their efficacy against pancreatic cancer [4–6]. However, the results of these trials are far from satisfactory [4–6]. It is therefore important to identify novel kinases that are vital for pancreatic cancer progression.

Phosphoenolpyruvate carboxykinase (PCK) is the first rate-limiting enzyme of gluconeogenesis that converts oxaloacetate and GTP into phosphoenolpyruvate (PEP) and CO_2_ [7]. It plays a vital role in gluconeogenesis [8]. There are two isoforms of PCK in mammals, including PCK1 and PCK2 [9]. *PCK1* gene locates at chromosome 20q13.31 and PCK1 protein mainly accumulates in the cytoplasm under unstimulated condition. Activated PCK1 can translocate into the endoplasmic reticulum [10].

Recent studies have reported upregulation and/or increased phosphorylation of PCK1 or PCK2 in different human malignancies, including colon cancer, non-small cell lung cancer (NSCLC), melanoma, and lymphoma, as well as metastatic breast cancer and hepatocellular carcinoma (HCC) [10–15]. These studies revealed that PCK1/2 could promote tumorigenesis and progression through non‐gluconeogenic mechanisms [10–15]. Xu et al., demonstrated that Akt-phosphorylated PCK1 can function as a protein kinase to phosphorylate insulin-induced gene 1 (INSIG1) and INSIG2 in HCC, thus promoting tumor growth [10, 15]. Targeting PCK1/2 could be novel therapeutic strategies to inhibit human cancers [10–15]. Here we show that PCK1 is required for pancreatic cancer cell growth both in vitro and in vivo.

## Materials and methods

### Chemicals and reagents

All sequences and viral constructs were provided by Shanghai Genechem Co. (Shanghai, China).

### Human tissues

Fresh pancreatic cancer tissues and adjacent normal pancreatic tissues from five primary pancreatic cancer patients (administrated at Affiliated Kunshan Hospital of Jiangsu University) were obtained. None of these patients received chemotherapy or radiotherapy before surgery. Written informed consent was obtained from each patient. The protocols were approved by the Ethics Board of Jiangsu University (BR2015021), according to the Declaration of Helsinki. The characteristic of the patients are summarized in Table 1.

**Table 1:** The clinicopathological features of pancreatic cancer patients.

No.	Gender	Age	Pathologic-T	Pathologic-N	Pathologic-M	Ki67 positive rate	AJCC stage
Patient-1	M	65	T4	N1	M1	60%	IV
Patient-2	F	72	T3	N2	M0	80%	III
Patient-3	F	40	T1c	N0	M0	1%	IA
Patient-4	M	67	T1c	N0	M0	10%	IA
Patient-5	M	48	T4	N0	M0	2%	III

### Mouse xenograft studies

Animal protocols have been approved by IACUC and the Ethics Review Board of Jiangsu University. Five-to six-week-old female BALB/c nude mice (18–19 g), purchased from the Animal Center of Soochow University, were raised indoors at standard conditions. PANC-1 cells (1 × 10^6^ cells per mouse, in 0.2 mL 10% FBS DMEM/Matrigel solution) with indicated genetic modifications were subcutaneously injected into the flanks of the nude mice. The mice body weights and tumor volumes were measured every 5 days with digital calipers [16]. The mice were sacrificed after 25 days.

All other methods were described in Supplement Information.

### Statistical analysis

The investigators were blinded to the group allocation during all in vitro experiments. In vitro experiments were repeated at least three times. Data with normal distribution were presented as mean ± standard deviation (SD). Statistical analysis was performed using SPSS 23.0 (SPSS Co., Chicago, IL). Unpaired student’s *t*-test and *χ*^2^ test were employed to compare two groups. One-way ANOVA with the Scheffe’ and Tukey Test was employed for comparison of more than two groups. *P* values of <0.05 were considered statistically significant.

## Results

### PCK1 is overexpressed in human pancreatic cancer tissues and cells

**Fig. 1:**
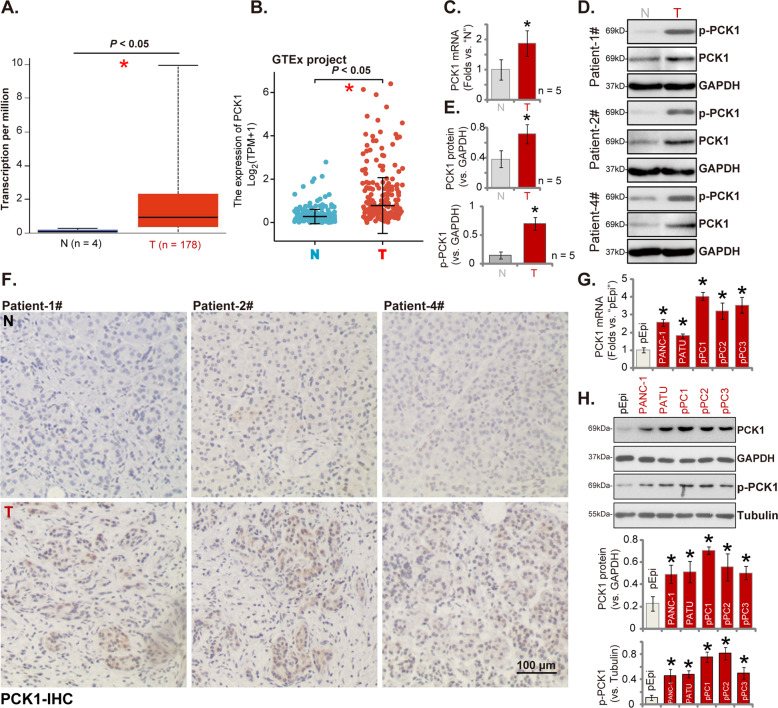
PCK1 is overexpressed in human pancreatic cancer tissues and cells. TCGA-PAAD cohorts show relative *PCK1 mRNA* expression in 171 cases of pancreatic ductal adenocarcinoma (PDAC) tissues (“T”) and four cases of normal pancreatic tissues (“N”) (**A**). GTEx project shows the RNA-Seq data of *PCK1* mRNA expression in primary pancreatic cancer tissues (“T”) and solid normal pancreatic tissues (“N”) (**B**). *PCK1* mRNA and listed proteins (total and phosphorylated PCK1) expression in five sets (*n* = 5) of pancreatic cancer tissues (“T”) and in normal tissues adjacent to tumor (“N”) from primary patients were tested by qRT-PCR (**C**). and Western blotting (**D**, **E**) assays, with results quantified. PCK1 immunohistochemistry (IHC) staining results of pancreatic cancer tissues and surrounding normal tissues from three representative patients (**F**). Expression of *PCK1* mRNA and listed proteins in listed pancreatic cancer cells and primary pancreatic epithelial cells (“pEpi”) was shown, with results quantified (**G**, **H**). PATU stands for “PATU-8988”. Data were presented as mean ± standard deviation (SD). *P < 0.05 vs. “N” tissues/“pEpi” cells. Scale bar = 100 μm (**F**).

First The Cancer Genome Atlas (TCGA) cohort was consulted to examine *PCK1* expression in pancreatic ductal adenocarcinoma (PDAC). As shown *PCK1* mRNA levels in pancreatic cancer tissues (“T”, *n* = 178) were significantly higher than those in normal pancreatic tissues (“N”, *n* = 4) (Fig. 1A). In addition, the GTEx project analyzing the RNA-Seq data of human cancers demonstrated that *PCK1* mRNA levels in pancreatic cancer tissues (“T”) were significantly higher than those in normal pancreatic tissues (“N”) (Fig. 1B).

To confirm the bioinformatics results, we examined PCK1 expression in local pancreatic cancer tissues. Human tissue specimens from five (*n* = 5) primary pancreatic cancer patients (Table 1) were obtained. The qRT-PCR assay results found that *PCK1* mRNA levels in pancreatic cancer tissues (“T”) were again significantly higher than those in adjacent normal tissues (“N”) (Fig. 1C). Western blotting assays were performed to test PCK1 protein expression and results confirmed PCK1 protein upregulation in pancreatic cancer tissues (“Patient #1/#2/#4”, three representative patients) (Fig. 1D). Increased PCK1 phosphorylation was detected as well. When combining the blotting data of all five sets of human tissues, we found that PCK1 protein and phosphorylation in pancreatic cancer tissues were significantly elevated (*P* < 0.05 vs. “N” tissues, Fig. 1E). IHC staining assay results further confirmed PCK1 protein upregulation in pancreatic cancer tissues in Patient #1/#2/#4 (Fig. 1F).

We also tested PCK1 expression in pancreatic cancer cells. Five different types of human pancreatic cancer cells were tested, including the established cell lines (PANC-1 and PATU-8988) as well as the primary human pancreatic cancer cells derived from three patients (namely “pPC1/pPC2/pPC3”). The qRT-PCR assay results, Fig. 1G, showed that *PCK1* mRNA expression levels were elevated in the pancreatic cancer cells when compared to low expression in primary pancreatic epithelial cells (“pEpi”) (Fig. 1G). PCK1 protein and phosphorylation were significantly elevated as well in the established and primary human pancreatic cancer cells (Fig. 1H).

### PCK1 silencing inhibits pancreatic cancer cell growth, proliferation, and motility

**Fig. 2:**
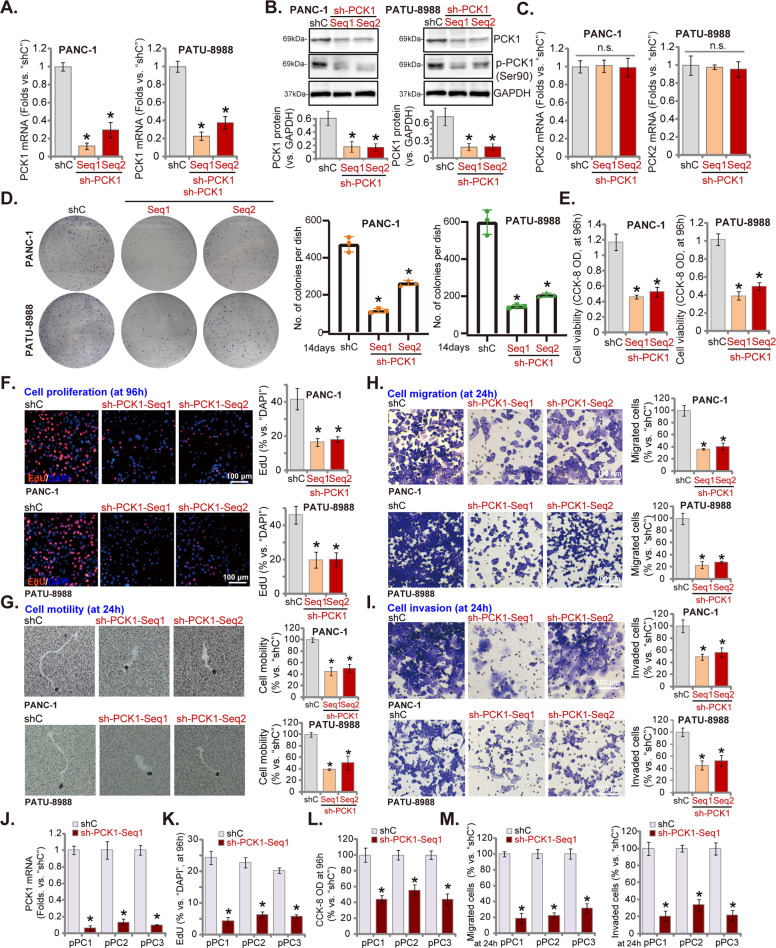
PCK1 silencing inhibits pancreatic cancer cell growth, proliferation, and motility. Established human pancreatic cancer cell lines (PANC-1 and PATU-8988) (**A**–**I**) or primary pancreatic cancer cells (“pPC1/2/3”) (**J**–**M**), bearing the PCK1 shRNA (“sh-PCK1-Seq1” or “sh-PCK1-Seq2”) or lentiviral scramble control shRNA (“shC”), were established. Expressions of *PCK1* mRNA (**A**, **J**) and protein (**B**), as well as *PCK2* mRNA (**C**) were shown; Cells were further cultured for applied time periods, colony formation (**D**), proliferation (**F**, **K**), viability (**E**, **L**), cell motility (**G**), cell migration and invasion (**H**, **I**, **M**) were tested by the listed assays mentioned in the text, with results quantified. For all EdU assays, five random views of total 2500 cell nuclei per treatment were included to calculate the average EdU ratio (% vs. DAPI). For all “Transwell” and “Matrigel Transwell” assays, five random microscopy views of each condition were included to calculate the average number of migrated/invaded cells. For all in vitro cellular functional studies, exact same number of viable cells with the applied genetic modifications was initially seeded (“Day-0”/“0 h”), and cells were cultured for applied time periods. Data were presented as mean ± standard deviation (SD, *n* = 5). *P < 0.05 vs. “shC” cells. “n.s.” stands for non-statistical difference (**C**). The experiments were repeated five times with similar results obtained. Scale bar = 100 μm (**F**–**I**).

To explore the potential function of PCK1 in pancreatic cancer cells, two lentiviral PCK1 shRNAs, with non-overlapping sequences (“sh-PCK1-Seq1/sh-PCK1-Seq2”), were individually transduced to PANC-1 cells and PATU-8988 cells. After selection by puromycin, stable cancer cells (PANC-1 and PATU-8988) bearing the PCK1 shRNA were established. The qRT-PCR assay results confirmed that *PCK1* mRNA levels were robustly decreased by PCK1 shRNA (Fig. 2A). PCK1 protein and phosphorylation levels were downregulated as well (Fig. 2B). *PCK2* mRNA expression was however unchanged by the applied PCK1 shRNAs (Fig. 2C).

Colony formation assay results, Fig. 2D, showed a significantly decreased number of viable PANC-1 cell colonies and PATU-8988 cell colonies after PCK1 silencing. Furthermore, CCK-8 assay results showed that PCK1 silencing led to significant viability reduction in PANC-1 cells and PATU-8988 cells (Fig. 2E). Evidenced by decreased EdU-positive nuclei ratio, PANC-1, and PATU-8988 cell proliferation was largely inhibited by the PCK1 shRNA (Fig. 2F). In addition, the phagokinetic track motility assay results confirmed that cell motility was significantly inhibited by shRNA-mediated knockdown of PCK1 in PANC-1 cells and PATU-8988 cells (Fig. 2G). Furthermore, testing cell migration through “Transwell” assays, we showed that pancreatic cell migration was decreased by the PCK1 shRNA (Fig. 2H). In addition, pancreatic cancer cell invasion, tested by “Matrigel Transwell” assays, was significantly attenuated as well (Fig. 2I).

In the primary pancreatic cancer cells derived from three patients, pPC1/pPC2/pPC3, the application of the lentiviral PCK1 shRNA (Seq1) led to robust *PCK1* mRNA silencing (Fig. 2J). Functional studies demonstrated that PCK1 shRNA inhibited cell proliferation (EdU-positive nuclei ratio decrease, Fig. 2K), viability (CCK-8 OD reduction, Fig. 2L), migration, and invasion (“Transwell” assays, results quantified in Fig. 2M) in primary cancer cells.

### PCK1 silencing provokes apoptosis activation in pancreatic cancer cells

**Fig. 3:**
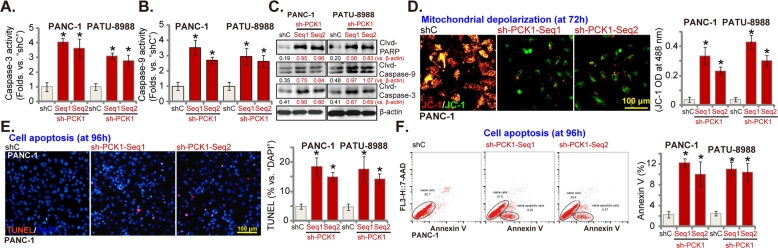
PCK1 silencing provokes apoptosis activation in pancreatic cancer cells. Established human pancreatic cancer cell lines (PANC-1 and PATU-8988) (**A**–**F**), bearing the PCK1 shRNA (“sh-PCK1-Seq1” or “sh-PCK1-Seq2”) or lentiviral scramble control shRNA (“shC”), were established and cultured for applied time periods. Caspase-PAPR activations were tested by mentioned assays (**A**–**C**); Mitochondrial depolarization was tested by JC-1 staining assays (**D**), with cell apoptosis examined by nuclear TUNEL staining (**E**), and Annexin V-FACS assays (**F**), with results quantified. Data were presented as mean ± standard deviation (SD, *n* = 5). *P < 0.05 vs. “shC” cells. The experiments were repeated five times with similar results obtained. Scale bar = 100 μm (**D**, **E**).

We next studied whether PCK1 silencing could provoke apoptosis activation in pancreatic cancer cells. In PANC-1 cells and PATU-8988 cells, following PCK1 silencing by targeted shRNAs (see Fig. 2), the caspase-3 activity (Fig. 3A) and the caspase-9 activity (Fig. 3B) were significantly increased. In addition, increased cleavages of caspase-3, caspase-9, and PARP were detected in cells with PCK1 shRNAs (Fig. 3C). In PCK1-silenced PANC-1 cells and PATU-8988 cells, JC-1 green monomers were formed, indicating mitochondrial depolarization (Fig. 3D). As shown in Fig. 3E, the TUNEL-positive nuclei ratio was significantly increased in PCK1-silenced PANC-1 cells and PATU-8988 cells, indicating apoptosis activation. FACS assay results, Fig. 3F, showed that PCK1 silencing led to significantly increased Annexin V ratio in pancreatic cancer cells, further supporting apoptosis activation.

### CRISPR/Cas9-mediated PCK1 knockout inhibits pancreatic cancer cell growth and induces apoptosis activation

**Fig. 4:**
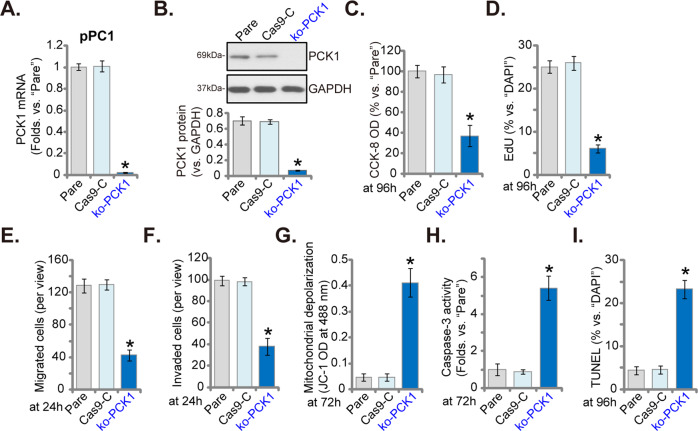
CRISPR/Cas9-mediated PCK1 knockout inhibits pancreatic cancer cell growth and induces apoptosis activation. The single stable primary pancreatic cancer cells (“pPC1”), bearing the lentiviral CRISPR/Cas9-PCK1-KO construct (“ko-PCK1”) or the CRISPR/Cas9 control empty vector (“Cas9-C”), as well as the parental control cells (“Pare”), were established; Expression of *PCK1* mRNA (**A**) and listed proteins (**B**) were tested. Cells were further cultured for applied time periods, cell viability (**C**), proliferation (**D**), migration, and invasion (**E**, **F**) were tested by the listed assays, with results quantified; Mitochondrial depolarization was tested by JC-1 intensity assay (**G**) and the caspase-3 activity was shown (**H**); Cell apoptosis was tested by nuclear TUNEL staining assays (**I**). Data were presented as mean ± standard deviation (SD, *n* = 5). *P < 0.05 vs. “Cas9-C” cells. The experiments were repeated five times with similar results obtained.

To further support the role of PCK1 in pancreatic cancer cells, a lentiviral CRISPR/Cas9-PCK1-KO construct was transduced to pPC1 primary pancreatic cancer cells. Single stable cells were established: ko-PCK1 cells (see Methods). As demonstrated *PCK1* mRNA (Fig. 4A) and protein (Fig. 4B) expressions were depleted in ko-PCK1 cells. In pPC1 cells, CRISPR/Cas9-induced PCK1 KO significantly decreased cell viability (CCK-8 OD, Fig. 4C) and inhibited cell proliferation (EdU-positive nuclei ratio reduction, Fig. 4D). Quantified results from “Transwell” (Fig. 4E) and “Matrigel Transwell” (Fig. 4F) assays confirmed that pPC1 cell migration and invasion were robustly inhibited with PCK1 KO.

Further studies demonstrated that JC-1 green monomer intensity increase, reflecting mitochondrial depolarization, was detected in PCK1 KO cells (Fig. 4G). In addition, when compared to the control cells expressing CRISPR/Cas9 empty vector (Cas9-C), the caspase-3 activity was significantly increased in the ko-PCK1 cells (Fig. 4H). Moreover, PCK1 KO in pPC1 cells induced significant apoptosis activation, which was evidenced by an increased TUNEL-positive nuclei ratio (Fig. 4I).

### Differentially expressed genes and altered signaling cascades in PCK1-silenced pancreatic cancer cells

**Fig. 5:**
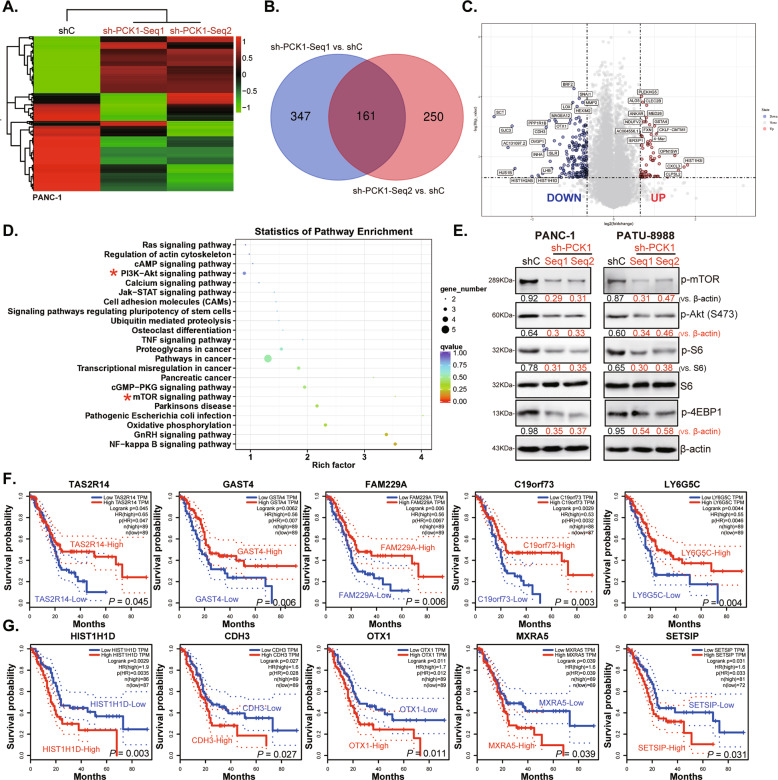
Differentially expressed genes and altered signaling cascades in PCK1-silenced pancreatic cancer cells. Heat map shows all differentially expressed genes (DEGs) in PANC-1 cells expressing the applied PCK1 shRNA (vs. cells with shC, **A**). Venn diagram shows 161 DGEs (both upregulated and downregulated genes) in PCK1 shRNA-expressing PANC-1 cells (**B**). Volcano plots show the representative DEGs in PCK1 shRNA-expressing PANC-1 cells (**C**). Signaling pathway analyses of DEGs performed with the KEGG and R software packages are shown (**D**). Western blotting was used to detect AKT-mTORC1 signaling activation in PANC-1 cells bearing the PCK1 shRNA (“sh-PCK1-Seq1” or “sh-PCK1-Seq2”) or lentiviral scramble control shRNA (“shC”) (**E**). TCGA Kaplan–Meier survival analyses according to expression levels of representative DEGs that were upregulated (**F**) and downregulated (**G**) in PCK1-silenced cells.

We next studied the possible underlying signaling mechanisms of PCK1-driven pancreatic cancer cell growth. High-throughput transcriptional profiling, or RNA-seq, was applied to analyze differentially expressed genes (DEGs) in PCK1-knockdown cells (Fig. 5A). As compared to PANC-1 cells with “shC”, 161 DEGs were screened out by plotting the Venn diagram in PANC-1 cells with “sh-PCK1-seq1” and “sh-PCK1-seq2” (Fig. 5B). The volcano map demonstrated the representative upregulated and downregulated DEGs in PCK1-silenced cells (Fig. 5C). Thereafter, a cluster profiler R package was utilized to examine the statistical enrichment of DEGs in KEGG pathways. Results showed that in PCK1-silenced PANC-1 cells DEGs are involved in the regulation of multiple signaling cascades (Fig. 5D). Among them, the phosphatidylinositol-3-kinase (PI3K)-Akt-mammalian cascade is one of the most significant one (“red stars”, Fig. 5D).

PI3K-Akt-mTOR activation is an extremely important signaling pathway for cell growth [17]. The abnormal activation of this cascade promotes pancreatic cancer tumorigenesis and progression [18–21]. Therefore, we analyzed whether PCK1 silencing shall affect this cascade in pancreatic cancer cells. Western blotting assay results, Fig. 5E, demonstrated that phosphorylations of mTOR, Akt, S6, and 4EBP1 were significantly decreased in PANC-1 cells and PATU-8988 cells with PCK1 shRNAs. Total protein levels of mTOR, Akt, S6, and 4EBP1 were unchanged (Fig. 5E).

To further support the role of PCK1 in Akt-mTOR activation, a PCK1-S90A was stably transduced to PATU-8988 cells. As shown, PCK1-S90A largely inhibited PCK1 Ser-90 phosphorylation (Fig. S1A). Consequently, phosphorylations of Akt and mTOR were significantly decreased in PATU-8988 cells (Fig. S1A). Importantly, PCK1-S90A inhibited PATU-8988 cell proliferation and migration, tested by nuclear EdU staining (Fig. S1B) and “Transwell” (Fig. S1C) assays, respectively.

Moreover, we integrated the mRNA expression of these DEGs with the clinical data from the TCGA database and divided the patients into high expression group and low expression group according to the median mRNA expression level. The R survival package was used for survival analyses. High expression of five representative DEGs that were upregulated in PCK1-silenced PANC-1 cells, including *TAS2R14*, *GAST4*, *FAM229A*, *C19orf73*, and *LY6G5C*, was significantly corrected with better overall survival (Fig. 5F). Conversely, high expression of five representative DEGs that were decreased in PCK1-silenced cells, including *IST1H1D*, *CDH3*, *OTX1*, *MXRA5*, and *SETSIP*, was significantly corrected with poor overall survival (Fig. 5G).

### Exogenous overexpression of PCK1 augments pancreatic cancer cell growth and motility

**Fig. 6:**
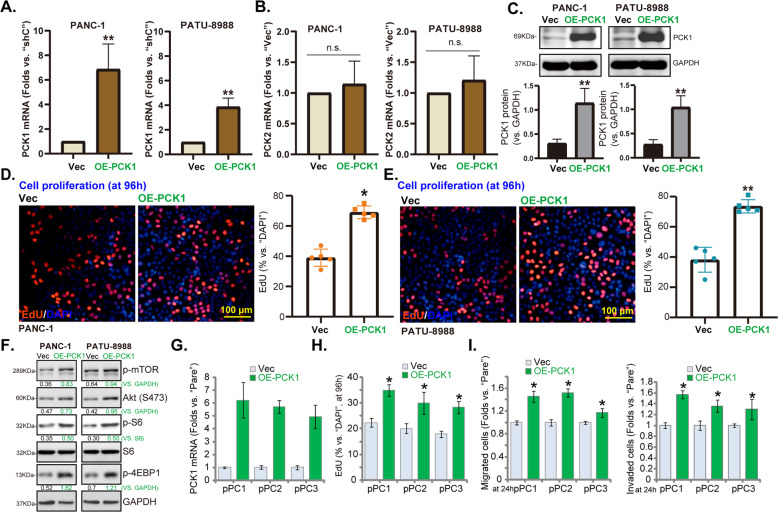
Exogenous overexpression of PCK1 augments pancreatic cancer cell growth and motility. Established pancreatic cancer cell lines (PANC-1 and PATU-8988) (**A**–**F**) or the primary pancreatic cancer cells (“pPC1/2/3”) (**G**–**I**) with a lentiviral construct encoding the PCK1 cDNA (“OE-PCK1”) or the empty vector (“Vec”), were established and cultured for applied time periods; Expression of listed mRNA and proteins were tested by qRT-PCR and Western blotting assays (**A**–**C**, **F**, **G**); Cell proliferation (EdU-positive nuclei ratio, **D**, **H**), migration and invasion (“Transwell” assays, **I**) were tested by the listed assays, with data quantified. Data were presented as mean ± standard deviation (SD, *n* = 5). *P < 0.05 vs. “Vec” cells. “n.s.” stands for non-statistical difference (**B**). The experiments were repeated five times with similar results obtained. Scale bar = 100 μm (**D**, **E**).

Next, a lentiviral construct encoding PCK1 cDNA (“OE-PCK1”) was transduced to PANC-1 cells and PATU-8988 cells. Stable cell lines were established through selection with puromycin. As compared to control cells with empty vector (“Vec”), *PCK1* mRNA levels in OE-PCK1 cells were significantly increased (Fig. 6A). PCK1 protein expression was upregulated as well in OE-PCK1 cells (Fig. 6B), where *PCK2* mRNA levels were unchanged (Fig. 6C). Functional studies demonstrated that the EdU-positive nuclei ratio was significantly increased in OE-PCK1 PANC-1 cells and PATU-8988 cells, suggest that PCK1 overexpression promoted cell proliferation (Fig. 6D, E). More importantly, Western blotting assay results showed that phosphorylations of mTOR, Akt, S6, and 4EBP1 were significantly enhanced in PCK1-overexpressed PANC-1 cells and PATU-8988 cells (Fig. 6F).

In the primary pancreatic cancer cells, pPC1/ pPC2/pPC3, stable transfection of the OE-PCK1 construct led to robust PCK1 mRNA upregulation (Fig. 6G). In these primary cancer cells, ectopic overexpression of PCK1 augmented cell proliferation (increased EdU-positive nuclei ratio, Fig. 6H), migration, and invasion (“Transwell assays”, results quantified in Fig. 6I). In PCK1-overexpressed (OE-PCK1) pPC1 cells and pPC2 cells, adding the mTORC1 inhibitor rapamycin, the Akt inhibitor MK-2206 [22], and the mTOR kinase inhibitor AZD2014 [23] potently inhibited cell proliferation (EdU-positive nuclei ratio reduction, Fig. S1D) and migration (Fig. S1E). These results further supported that Akt-mTOR activation could be a key mechanism of PCK1-driven pancreatic cancer cell progression.

### PCK1 silencing suppresses pancreatic cancer xenograft growth in mice

**Fig. 7:**
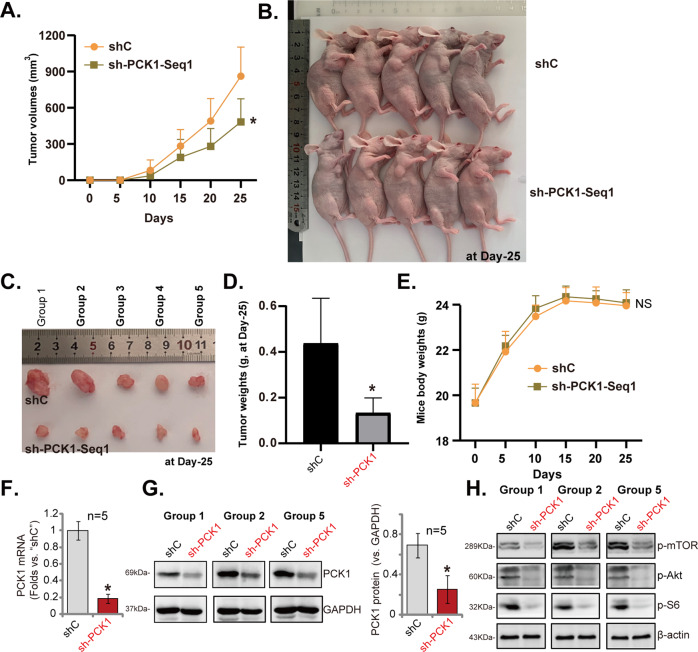
PCK1 silencing suppresses pancreatic cancer xenograft growth in mice. PANC-1 xenografts-bearing female BALB/c nude mice were established by subcutaneous injection of PANC-1 cells expressing PCK1 shRNA (“sh-PCK1-seq1”) or scramble control shRNA lentivirus (“shC”); Tumor volumes (**A**) and mice body weights (**E**) were recorded every five days. After 25 days, all tumors were separated (**B**, **C**) and tumor weights were recorded (**D**). In tumor tissue lysates expressions of *PCK1* mRNA (**F**) and listed proteins (**G**, **H**) were tested by qRT-PCR and Western blotting assays, with results quantified. Data were presented as mean ± standard deviation (SD). *P < 0.05 vs. “shC” tumors.

To test whether PCK1 can exert similar tumor-promoting activity in vivo, we utilized a xenograft mouse model. The equal amount of PANC-1 cells, bearing PCK1 shRNA (“sh-PCK1-seq1”) or scramble control shRNA (“shC”), were subcutaneously (s.c.) injected into the flanks of the nude mice (five mice per group, *n* = 5). Tumor growth curve results, Fig. 7A, demonstrated that PANC-1 xenografts-bearing PCK1 shRNA grew significantly slower than control xenografts with shC. PANC-1 xenograft tumors were removed 25 days after injection of PANC-1 cells (Fig. 7B). As shown PCK1 shRNA-expressing PANC-1 xenografts were significantly smaller (Fig. 7C) and lighter (Fig. 7D) than control PANC-1 xenografts expressing shC. These results further supported that PCK1 shRNA inhibited PANC-1 xenograft growth in mice. The mice body weights were not significantly different between the two groups (Fig. 7E).

Part of the tumor xenografts were lysed in fresh tumor tissue lysates. The qRT-PCR assay results confirmed robust *PCK1* mRNA reduction in PCK1 shRNA-expressing PANC-1 xenograft tissues (Fig. 7F). Results in Fig. 7G further confirmed PCK1 protein silencing. Moreover, phosphorylations of mTOR, Akt, and S6 were significantly decreased in PCK1 shRNA-expressing PANC-1 xenograft tissues (Fig. 7H).

## Discussion

Emerging studies have revealed that metabolic reprogramming is a typical characteristic of cancers [24]. The nutritional and anabolic components of tumor cells provided by metabolic reprogramming are essential to maintain proliferative characteristics and to meet energy requirements [25–27]. Metabolic pathways, including glucose metabolism, citric acid (TCA) circulation, and lipogenesis, could support macromolecular synthesis in cancer cells to a large extent [28]. PCK1 is the rate-limiting enzyme of gluconeogenesis [7]. It plays an important role in metabolic reprogramming [8].

Recent studies have proposed a pivotal function of PCK1 in human cancer tumorigenesis progression [8, 10, 15, 29, 30]. Most of these studies proposed the cancer-promoting function of PCK1 in different types of cancer. Yamaguchi et al. demonstrated that PCK1 overexpression increased glucose consumption and promoted colon cancer cell proliferation [31]. Xu et al. found that PCK1 silencing inhibited phosphorylation of INSIG1/2, thus decreasing proliferation of HCC cells and tumorigenesis in mice [10, 15]. In NSCLC, PCK1-induced nuclear SCAP-sterol regulatory element-binding protein 1 (SREBP1) activation is required for cancer progression [29]. PCK1 was also reported to augment CRC liver metastatic growth by driving pyrimidine nucleotide biosynthesis under hypoxia conditions [30]. Li et al. reported that PCK1 promoted the growth of melanoma TRCs (tumor-repopulating cells). TRCs transduced extracellular signaling by αVβ3 integrin, leading to the activation of PI3K and histone methylation, which will further regulate PCK1 expression [12]. Other studies, however, proposed a potential tumor-suppressive function of PCK1. Liu et al. reported that PCK1 promoted TCA cataplerosis, oxidative stress, and apoptosis in liver cancer cells [8]. Tuo et al. found that PCK1 silencing can accelerate hepatoma cell growth by activating the Nrf2 signaling cascade [32]. Hence, it is currently not clear how PCK1 expression leads to such contrasting consequences in different tumors.

In this study, our results suggest that PCK1 could be an important gene for pancreatic cancer cell growth. PCK1 is overexpressed in pancreatic cancer tissues. PCK1 upregulation is also detected in established and primary human pancreatic cancer cells. Its expression is relatively low in pancreatic epithelial cells. In pancreatic cancer cells, shRNA-induced PCK1 silencing or CRISPR/Cas9-induced PCK1 KO robustly inhibited cell growth, viability, proliferation, migration and invasion, and provoked apoptosis activation. Conversely, ectopic overexpression of PCK1 in established and primary pancreatic cancer cells augmented cell proliferation and mobility. In vivo, the growth of PANC-1 xenografts in SCID mice was largely inhibited after PCK1 silencing.

PCK1 kinase activity promoted SREBP1-dependent lipogenesis to promote HCC growth and tumorigenesis [10]. PCK1 pS90 was upregulated in esophageal carcinoma, correlating with poor prognosis [33]. Yamaguchi et al. reported that PCK1 augmented liver metastatic growth by promoting pyrimidine nucleotide biosynthesis under hypoxia [30]. PI3K-Akt-mTOR signaling cascade is frequently dysregulated and overactivated in pancreatic cancer, serving as an important etiology of the disease [20, 21]. Hyperactivation of this cascade is often associated with poor prognosis, as it critically regulates cell metabolism and proliferation, cell cycle progression, and protein synthesis, as well as cell survival, apoptosis resistance, and genomic instability [20, 21].

Here we found that PCK1 is important for Akt-mTOR activation in pancreatic cancer cells. RNA-seq analyzing DEGs in PCK1-silenced PANC-1 cells showed that DEGs are enriched in PI3K-Akt-mTOR cascades. Importantly, in PANC-1 and PATU-8988 cells, Akt-mTOR activation was largely inhibited by shRNA-induced silencing of PCK1 but was augmented after ectopic PCK1 overexpression. Furthermore, Akt-mTOR inactivation was detected in PCK1 shRNA-expressing PANC-1 xenograft tumor tissues. Moreover, the PCK1-S90A suppressed Akt-mTOR activation and inhibited pancreatic cancer migration and proliferation. These results implied a pivotal role of PCK1 in the activation of PI3K-Akt-mTOR cascade in pancreatic cancer cells. The underlying signaling mechanisms of PCK1-driven pancreatic cancer growth may warrant further characterizations. The pathological mechanisms of increased PCK1 expression and phosphorylation in pancreatic cancer require further investigations as well. Although our preliminary findings implied a possible role of microRNA dysregulation in the process.

## Conclusion

PCK1 is a potential therapeutic target for pancreatic cancer.

## Supplementary information

Supplementary Methods

Figure S1

## Data Availability

All data are available upon request.
