# Supplementary material for: Identification of phosphoenolpyruvate carboxykinase 1 as a potential therapeutic target for pancreatic cancer
Source: Cell Death Dis. 2021 Oct 7;12(10):918. doi: 10.1038/s41419-021-04201-w (PMC8497628; doi:10.1038/s41419-021-04201-w)
Supplement: Supplementary file 1 — Supplementary Methods [file 41419_2021_4201_MOESM1_ESM.docx]

**Supplement Information**

**Cell culture.** Pancreatic cancer cell lines, PANC-1 and PATU-8988, were provided by Dr. Cao at Soochow University [^1^](#_ENREF_1)^,^ [^2^](#_ENREF_2). Cells were cultured in high glucose (17.51 mM) DMEM/F-12 medium plus 10% FBS (Gibco, Suzhou, China), in a incubator with humidified atmosphere of 5% CO_2_. For primary cell culture, pancreatic cancer tissue specimens from three written-informed consent patients,“pPC1/ pPC2/pPC3”, undergoing cancer resection were freshly restored in liquid nitrogen. Tissues were washed, minced, and digested via Collagenase I and DNase (Sigma). After incubation, the mixture was centrifuged at 2000g for 3 min prior to suspension in fresh media. The cell suspensions were immediately plated onto cell chambers and cultured in high glucose (17.51 mM) DMEM/F-12 medium plus 12.5% FBS. Fibroblasts, blood vessel cells and immune cells in the medium supernatant were immediately removed after cancer cell attachment. Cancer cells were verified by K-RAS mutation. Same procedure to applied to surrounding normal pancreatic tissues to primary culture pancreatic epithelial cells (“pEpi”). Primary cells were utilized for 8-10 generations [^3^](#_ENREF_3). Cells were always starved in low-serum (1.5 % FBS) high glucose (17.51 mM) DMEM/F-12 medium overnight before *in vitro* experiments. The protocols were approved by the Ethics Committee of Jiangsu University, in accordance to the Declaration of Helsinki. Cells were subjected to mycoplasma and microbial contamination examination. STR profiling, population doubling time, morphology, as well as histology of the cells were examined to verify the genotypes.

**Bioinformatics analysis.** Gene Expression Profiling Interactive Analysis (GEPIA) database was consulted to analyze PCK1 gene expression profile for pancreatic cancer patients from the TCGA data portal. The Genotype-Tissue Expression project (GTEx) was utilized to analyze the RNA-Seq data of PCK1 for pancreatic cancer tissues and solid normal pancreatic tissues.

**Antibodies.** The primary antibodies utilized were as follows: anti-PCK1 (1:1000; #ab133603, Abcam), anti-β-actin (1:20, 000; #ab8227, Abcam), anti-GAPDH (1:2000; #51332, Cell Signaling Technology), anti-poly (ADP-ribose) polymerase (PARP, 1:1000; #9542,Cell Signaling Technology), anti-cleaved-PARP (E51) (1:1000; #ab32064, Abcam), anti-cleaved-Caspase9 (1:1000; #ab2324, Abcam), anti-cleaved-Caspase3 (1:1000; # ab2302, Abcam), anti-p-mTOR (Ser2448), 1:1000; #ab109628, Abcam), anti-phospho-Akt (S473) (1:1000; #4060, Cell Signaling Technology), anti-S6 (1:1000; #2217, Cell Signaling Technology), anti-phospho-S6 (1:1000; #ab12864, Abcam), anti-phospho-4EBP1 (1:1000, #9456, Cell Signaling Technology).The anti-phospho-PCK1 (Ser-90) antibody (#58006) was from SignalAntibody (Shanghai, China).

**PCK1 shRNA.** Two different lentiviral PCK1 shRNAs (in GV493hU6-MCS-CBh-IRES-puromycin vector), targeting non-overlapping sequences of PCK1 (“sh-PCK1-Seq1/ sh-PCK1-Seq2"), were synthesized and verified by Genechem (Shanghai, China). Pancreatic cancer cells were seeded into six-well plates at 50% confluence. Cells were then transduced with PCK1 shRNA lentivirus for 24h and stable cells were selected by puromycin (2 µg/mL, for five passages). PCK1 silencing in the stable cells was verified by qRT-PCR and Western blotting assays.

**Ectopic overexpression of PCK1.** The full-length wild-type PCK1 or phosphorylation-deficient mutant PCK1 (S90A, based on the described protocol [^4^](#_ENREF_4)) were synthesized by Genechem (Shanghai, China). Each was sub-cloned into a GV492 gene expression lentiviral vector (Ubi-MCS-3FLAG-CBh-IRES-puromycin, Genechem) to generate PCK1-expressing lentiviral construct. Pancreatic cancer cells were seeded into six-well plates (at 50% confluence). The construct was transduced to pancreatic cancer cells, and stable cells selected by puromycin (2 µg/mL). PCK1 overexpression in the stable cells was verified by qRT-PCR and Western blotting assays.

**PCK1 knockout.** A lentiviral CRISPR/Cas9-PCK1-KO construct was designed, assembled and verified by Shanghai Genechem Co. (Shanghai, China). The targeted DNA sequence is CGTTCAATGCCAGGTTCCCA with PAM sequence GGG. The construct was transduced to pancreatic cancer cells (cultured at 50% confluence). Cells were then distributed into 96 well plates. In each well cells were subjected to PCK1-KO screening. Single stable PCK1-KO cells were then established and were named as “ko-PCK1” cells. PCK1 KO was further verified by qRT-PCR and Western blotting assays.

**Quantitative real-time reverse transcriptase polymerase chain reaction** **(qRT-PCR) assay.** The detailed protocols were described in our previous studies [^5^](#_ENREF_5)^,^ [^6^](#_ENREF_6). In brief, TRIzol reagents were added to cultured cells or fresh tissue specimens to obtain total RNA, which was reversely transcripted to cDNA. Through an ABI7600 Prism system, qRT-PCR was performed through a SYBR Green PCR kit. Melt curve analysis was always performed to calculate the product melting temperature. The 2^-∆∆Ct^ method was used for the quantification of targeted mRNA, with *GAPDH* mRNA tested as an internal control. The mRNA primers were synthesized and verified by Genechem.

**Western blotting.** In brief, aliquots of 20μg protein from each sample were separated by 10-12% SDS-polyacrylamide gel electrophoresis (SDS-PAGE) and transferred to a polyvinylidene difluoride (PVDF) membrane (Millipore, Bedford, MA). Detailed protocols of Western blotting assays were reported early [^6^](#_ENREF_6)^,^ [^7^](#_ENREF_7)^,^ [^8^](#_ENREF_8)^,^ [^9^](#_ENREF_9). The data quantification was through an ImageJ software.

**Cell viabilityCCK-8 dye assay.** Cells (2 × 10^3^ cells per well) were seeded into 96-well plates and cultured for applied time periods. Afterwards, 10 μL CCK-8 reagent was added into each well and incubated for another 3h. CCK-8 optical density (OD) value was recorded at 450 nm.

**EdU (5-ethynyl-20-deoxyuridine) incorporation.** Cells were seeded into 24-well tissue culture plates (at 5 × 10^4^ cells per well). EdU Apollo-567 In Vitro Imaging Kit (Ribo-Bio, Guangzhou, China) was utilized to measure cell proliferation according to the manufacturer’s protocol. EdU (50 μM) was added to each well for 4h. Cell nuclei were co-stained with DAPI and were visualized under a fluorescent microscope (OLYMPUS, Japan).

**Colony formation.** Pancreatic cancer cells with applied genetic modifications were seeded into six-well plates at 1, 500 cells per well. Cells were cultured for 14 days. Afterwards, cells were fixed and stained with 2.5% crystal violet. The plates were washed and viable cell colonies were manually counted.

**Cell migration, invasion and phagokinetic track motility assays.** *In vitro* cell migration assays were performed by using “Transwell” chambers (8 µm pore size, Corning, Shanghai, China). Cells with the applied genetic modifications were resuspended in serum-free medium and added to the surface of chambers. For each chamber 600 µL DMEM containing 10 % FBS was added to the lower compartment. After incubation for 24h, cells located on the upper surfaces were gently scraped using cotton swabs. The lower chamber membranes were fixed with 4% paraformaldehyde and stained with 2.5% crystal violet. For invasion assays, “Transwell” chambers were always pre-coated with Matrigel (Sigma). The detailed protocols of phagokinetic track motility assay were described in elsewhere [^10^](#_ENREF_10)^,^ [^11^](#_ENREF_11).

**Caspase activity**. Caspase-3 and caspase-9 activities were measured by using a commercial kit (ab219915; Abcam). Briefly, pancreatic cancer cells with applied genetic modifications were seeded into 96-well plates (4 × 10^3^ cells per well) and cultured for applied time periods. Cells were then incubated with 10 × test compounds and loading solution (containing the assay buffer and the caspase-3/-9 substrate) for 1 h. Then, the caspase activity was tested by a fluorescence microplate reader (BioTek Synergy), and 620 nm emission for caspase-3 and 450 nm emission for caspase-9.

**Cell apoptosis assays.** Cells with applied treatments were re-suspended and stained with Annexin V-PE (10 μg/mL) and 7-AAD or propidium Iodide (PI) (10 μg/mL, BD Bioscience, Shanghai, China) and were detected by FACS using a Becton-Dickinson Flow Cytometer (BD Bioscience, Shanghai, China). Annexin V-positive cells were labeled as apoptotic cells. Other cell apoptosis assays, including JC-1 assay of mitochondrial depolarization, nuclear TUNEL (TdT mediated dUTP Nick End Labeling) staining assay of cell apoptosis were described in our previous studies [^9^](#_ENREF_9)^,^ [^12^](#_ENREF_12)^,^ [^13^](#_ENREF_13).

**Transcriptome sequencing analysis (RNA sequencing).** For sample collection and preparation, RNA integrity was assessed by the RNA Nano 6000 assay kit. Total RNA was analyzed by the Bioanalyzer 2100 system (Agilent Technologies, CA). A total amount of 1 μg RNA of each sample was utilized as input material. mRNA was purified from total RNA using poly-T oligo-attached magnetic beads. The fist strand cDNA was synthesized using random hexamer primer and M-MuLV Reverse Transcriptase (RNase H-) while second strand cDNA synthesis was subsequently performed using DNA Polymerase I and RNase H. The library preparations were sequenced on an Illumina Novaseq platform and 150 bp paired-end reads were generated. For data analyses, reference genome and gene model annotation files were downloaded from genome website. Index of the reference genome was built and paired-end clean reads were aligned to the reference genome using the Hisat2 v2.0.5. Differential expression analysis of three groups (two biological replicates per condition) was performed using the DESeq2-R package (1.20.0). Differential genes in the samples were analyzed. KEGG is a database resource for understanding high-level functions and utilities of the biological system[^14^](#_ENREF_14).^.^We utilized cluster Profiler R package to test the statistical enrichment of differential expression genes in KEGG pathways. Finally, overall survival analysis (OS) of representative DGEs in pancreatic cancer patients of TCGA database was analyzed by R survival package.

**Immunohistochemistry (IHC).** IHC staining was performed on 4% paraformaldehyde-fixed, paraffin-embedded tissue sections (3 μm) according to standard procedures. Briefly, tumor slides were incubated anti-PCK1 (1: 25, Abcam) and were subsequently stained with corresponding secondary antibody (Santa Cruz). The peroxidase activity was visualized using a 3-amino-9-ethyl-carbazol (AEC) method (Merck, Shanghai, China). All tissue sections were scored by the semi-quantitative H-score approach and validated by two experienced pathologists.

**References**

1. Xu, X. D., Yang, L., Zheng, L. Y., Pan, Y. Y., Cao, Z. F., Zhang, Z. Q., et al. Suberoylanilide hydroxamic acid, an inhibitor of histone deacetylase, suppresses vasculogenic mimicry and proliferation of highly aggressive pancreatic cancer PaTu8988 cells. *BMC Cancer.* **14**, 373 (2014).

2. Wu, C. H., Cao, C., Kim, J. H., Hsu, C. H., Wanebo, H. J., Bowen, W. D., et al. Trojan-horse nanotube on-command intracellular drug delivery. *Nano Lett.* **12**, 5475-5480 (2012).

3. Minjie, S., Defei, H., Zhimin, H., Weiding, W. & Yuhua, Z. Targeting pancreatic cancer cells by a novel hydroxamate-based histone deacetylase (HDAC) inhibitor ST-3595. *Tumour Biol.* **36**, 9015-9022 (2015).

4. Xu, D., Wang, Z., Xia, Y., Shao, F., Xia, W., Wei, Y., et al. The gluconeogenic enzyme PCK1 phosphorylates INSIG1/2 for lipogenesis. *Nature.* **580**, 530-535 (2020).

5. Bai, J. Y., Li, Y., Xue, G. H., Li, K. R., Zheng, Y. F., Zhang, Z. Q., et al. Requirement of Galphai1 and Galphai3 in interleukin-4-induced signaling, macrophage M2 polarization and allergic asthma response. *Theranostics.* **11**, 4894-4909 (2021).

6. Liu, Y. Y., Chen, M. B., Cheng, L., Zhang, Z. Q., Yu, Z. Q., Jiang, Q., et al. microRNA-200a downregulation in human glioma leads to Galphai1 over-expression, Akt activation, and cell proliferation. *Oncogene.* **37**, 2890-2902 (2018).

7. Zheng, J., Zhang, Y., Cai, S., Dong, L., Hu, X., Chen, M. B., et al. MicroRNA-4651 targets bromodomain-containing protein 4 to inhibit non-small cell lung cancer cell progression. *Cancer Lett.* **476**, 129-139 (2020).

8. Xu, M., Wang, Y., Zhou, L. N., Xu, L. J., Jin, Z. C., Yang, D. R., et al. The therapeutic value of SC66 in human renal cell carcinoma cells. *Cell Death Dis.* **11**, 353 (2020).

9. Wang, S. S., Lv, Y., Xu, X. C., Zuo, Y., Song, Y., Wu, G. P., et al. Triptonide inhibits human nasopharyngeal carcinoma cell growth via disrupting Lnc-RNA THOR-IGF2BP1 signaling. *Cancer Lett.* **443**, 13-24 (2019).

10. Cao, C., Huang, X., Han, Y., Wan, Y., Birnbaumer, L., Feng, G. S., et al. Galpha(i1) and Galpha(i3) are required for epidermal growth factor-mediated activation of the Akt-mTORC1 pathway. *Sci Signal.* **2**, ra17 (2009).

11. Cao, C., Sun, Y., Healey, S., Bi, Z., Hu, G., Wan, S., et al. EGFR-mediated expression of aquaporin-3 is involved in human skin fibroblast migration. *Biochem J.* **400**, 225-234 (2006).

12. Liu, Z., Li, P., Yang, Y. Q., Cai, S., Lin, X., Chen, M. B., et al. I-BET726 suppresses human skin squamous cell carcinoma cell growth in vitro and in vivo. *Cell Death Dis.* **11**, 318 (2020).

13. Chen, M. B., Liu, Y. Y., Xing, Z. Y., Zhang, Z. Q., Jiang, Q., Lu, P. H., et al. Itraconazole-Induced Inhibition on Human Esophageal Cancer Cell Growth Requires AMPK Activation. *Mol Cancer Ther.* **17**, 1229-1239 (2018).

14. Doncheva, N. T., Palasca, O., Yarani, R., Litman, T., Anthon, C., Groenen, M. A. M., et al. Human pathways in animal models: possibilities and limitations. *Nucleic Acids Res.* **49**, 1859-1871 (2021).
