# Supplementary material for: Identification of phosphoenolpyruvate carboxykinase 1 as a potential therapeutic target for pancreatic cancer
Source: Cell Death Dis. 2021 Oct 7;12(10):918. doi: 10.1038/s41419-021-04201-w (PMC8497628; doi:10.1038/s41419-021-04201-w)
Supplement: Supplementary file 2 — Figure S1 [file 41419_2021_4201_MOESM2_ESM.pdf]

# Figure S1

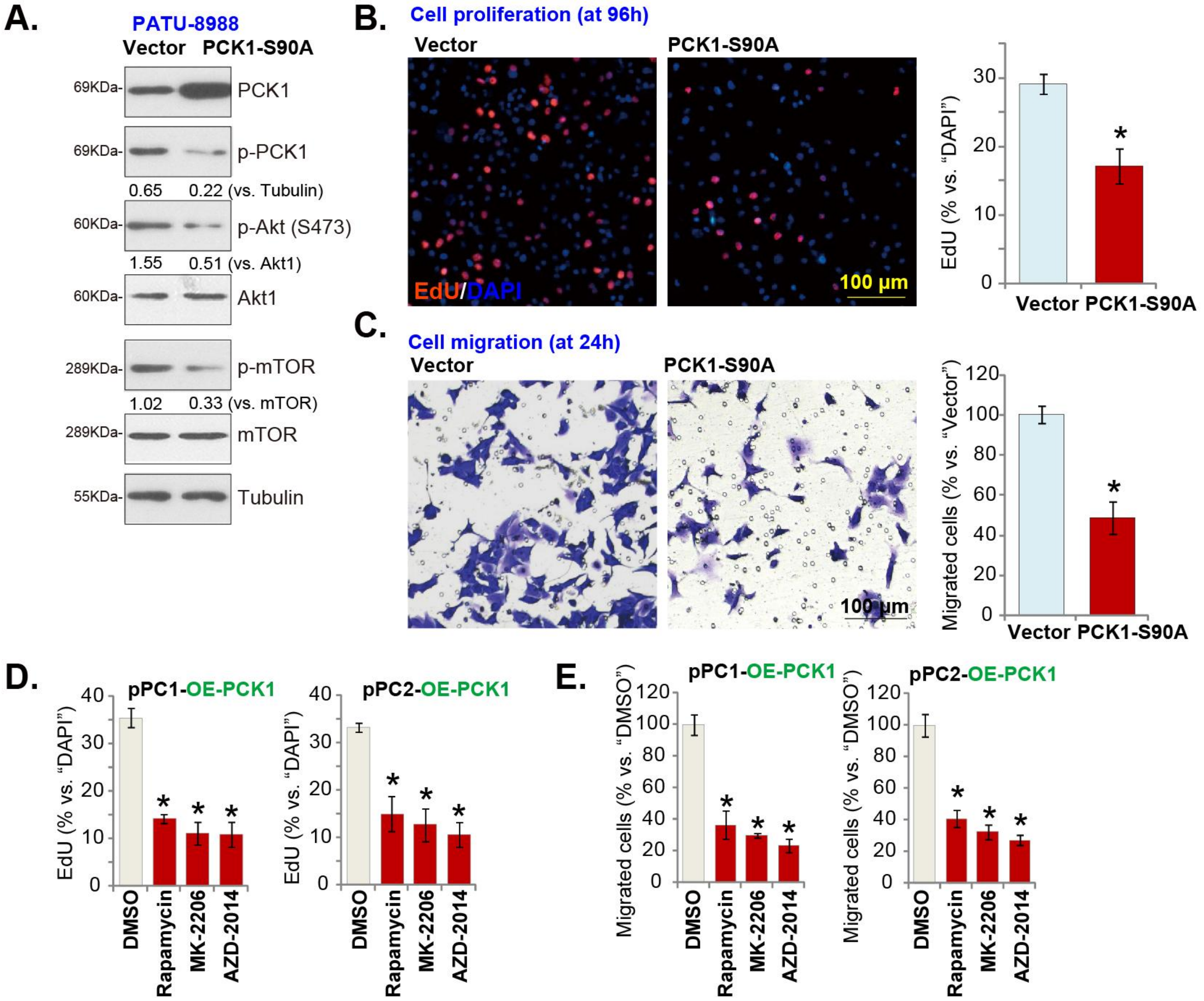


**Figure S1**. PATU-8988 cells with lentiviral construct encoding the PCK1-S90A or the empty vector ("Vec") were established; Expression of listed proteins was tested by Western blotting assays (**A**); Cells were further cultured for applied time periods, cell proliferation (EdU-positive nuclei ratio, **B**) and migration ("Transwell" assays, **C**) were tested, and data quantified. The primary pancreatic cancer cells ("pPC1 and pPC2 ") with lentiviral construct encoding the PCK1 cDNA ("OE-PCK1") were treated with 250 nM of rapamycin, MK-2206 or AZD2014 for applied time periods, cell proliferation (EdU-positive nuclei ratio, **D**), and migration (by recording migrated cell number, **E**) were tested, with data quantified. Expression of listed proteins was quantified (**A**). Data were presented as mean ± standard deviation (SD, n=5). * ***P*** < 0.05 vs. "Vector" cells (**B** and **C**). * ***P*** < 0.05 vs. vehicle control (0.1% DMSO) group (**D** and **E**). Data were presented as mean ± standard deviation (SD, n=5). Scale bar = 100 μm (**B** and **C**).
